# Reconsidering arm positioning during pediatric general anesthesia based on natural sleep posture

**DOI:** 10.1186/s40981-026-00854-8

**Published:** 2026-03-25

**Authors:** Keisuke Yoshida, Yuko Matsumoto, Tatsumi Yakushiji, Takayuki Hasegawa, Satoki Inoue

**Affiliations:** 1 Department of Anesthesiology, Fukushima Medical University School of Medicine, Fukushima, Fukushima Japan; 2 Department of Anesthesiology, Saitama Medical Center, Kawagoe, Saitama Japan

To the Editor,

Proper patient positioning during general anesthesia is crucial because inappropriate positioning is associated with perioperative peripheral nerve injuries. In the supine position, abduction of the upper extremities beyond 90 degrees has been shown to increase the risk of brachial plexus injury because it may involve hyperextension or excessive external rotation of the shoulders [1, 2]. These findings are derived entirely from adult data; consequently, no explicit guidance have yet been established regarding cephalad upper limb positioning during routine general anesthesia in young children, whose anatomical and physiological characteristics differ substantially from those of adults [3].

Young children, particularly those aged between zero and five years, often sleep with their arms raised near or above the head (Fig. 1a). This observation suggests that raising both arms may represent a physiologically relaxed position in younger children. Therefore, caution is warranted in extrapolating adult-derived positioning practices to pediatric patients. On the basis, we suggest that, in the supine position under general anesthesia, allowing both arms to be positioned cephalad may be more appropriate for children. This proposal may also have educational value for daily pediatric anesthesia practice.

**Fig. 1:**
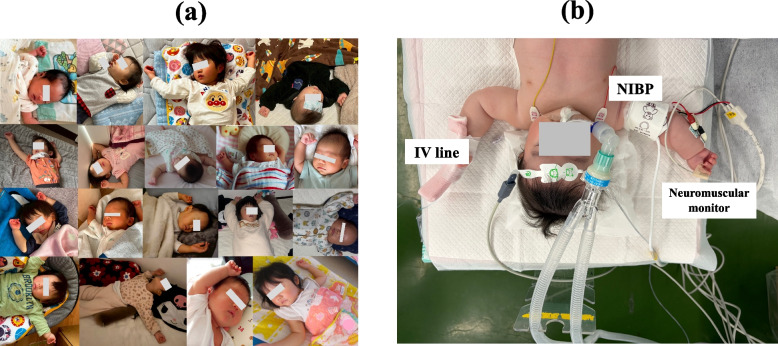
**a** A composite photograph showing natural sleep postures of children aged 0–5 years, with both upper limbs raised toward the head and the elbows slightly flexed. **b** A four-month-old infant after the induction of general anesthesia. The upper limbs are raised toward the head in a naturally relaxed position (up to the level of the child’s ears), abducted approximately 90–110 degrees, with the elbows slightly flexed. An intravenous line is placed on the dorsum of the left hand, and a neuromuscular monitor is attached at the right ulnar site. This position also makes it easier to check the strength of the radial pulse if NIBP fails or yields unreliable values, which frequently occurs in children. *NIBP: non-invasive blood pressure*

Positioning both arms cephalad during pediatric anesthesia offers several potential advantages. In pediatric surgery, it is often difficult to check or secure intravenous or arterial lines because the surgical or sterile field frequently overlaps the insertion sites, owing to the patient’s small body size [4]. When insertion sites such as the dorsal hand veins or radial arteries are located closer to the head, visualizing and securing additional vascular access become easier. This position may also facilitate neuromuscular monitoring via the ulnar nerve and the evaluation of capillary refill time [5]. Furthermore, placing infusion lines closer to the head shortens the tubing, enabling faster adjustment of infusion rates, thereby producing more immediate pharmacodynamic responses. Shorter tubing also reduces the discard volume needed before arterial blood sampling.

Nevertheless, several precautions should be considered. There has been a reported case in which neonates developed upper limb paresis following excessive elevation of the arms during surgery [6]. Observations of natural sleep posture in infants indicate that shoulder abduction of approximately 90–120 degrees with the elbows flexed to about 90 degrees appears to be a comfortable and natural position (Fig. 1b). Therefore, such a range of shoulder abduction may be considered when determining arm positioning during general anesthesia in infants. Although children generally have greater shoulder mobility, excessive external rotation should still be avoided. Clinical studies on upper limb positioning during general anesthesia in children are challenging because mild neurological symptoms are difficult to detect and because their anatomical and physiological characteristics exhibit considerable heterogeneity [3]. Unlike during natural sleep, when infants can spontaneously adjust their limb position or turn over in response to discomfort, limb position during general anesthesia cannot be spontaneously adjusted. Although infants may at times remain in a similar posture for a period of time during natural sleep with their arms elevated, this observation should not be interpreted as defining a duration that guarantees safety during general anesthesia. In practice, this positioning may be less concerning during shorter procedures; however, increased vigilance is warranted as operative duration increases.

## Data Availability

Not applicable.
